# A simple method for defining malaria seasonality

**DOI:** 10.1186/1475-2875-8-276

**Published:** 2009-12-03

**Authors:** Arantxa Roca-Feltrer, Joanna RM Armstrong Schellenberg, Lucy Smith, Ilona Carneiro

**Affiliations:** 1 Department of Infectious and Tropical Diseases, London School of Hygiene and Tropical Medicine, Keppel Street, London WC1E 7HT, UK

## Abstract

**Background:**

There is currently no standard way of defining malaria seasonality, resulting in a wide range of definitions reported in the literature. Malaria cases show seasonal peaks in most endemic settings, and the choice and timing for optimal malaria control may vary by seasonality. A simple approach is presented to describe the seasonality of malaria, to aid localized policymaking and targeting of interventions.

**Methods:**

A series of systematic literature reviews were undertaken to identify studies reporting on monthly data for full calendar years on clinical malaria, hospital admission with malaria and entomological inoculation rates (EIR). Sites were defined as having 'marked seasonality' if 75% or more of all episodes occurred in six or less months of the year. A 'concentrated period of malaria' was defined as the six consecutive months with the highest cumulative proportion of cases. A sensitivity analysis was performed based on a variety of cut-offs.

**Results:**

Monthly data for full calendar years on clinical malaria, all hospital admissions with malaria, and entomological inoculation rates were available for 13, 18, and 11 sites respectively. Most sites showed year-round transmission with seasonal peaks for both clinical malaria and hospital admissions with malaria, with a few sites fitting the definition of 'marked seasonality'. For these sites, consistent results were observed when more than one outcome or more than one calendar year was available from the same site. The use of monthly EIR data was found to be of limited value when looking at seasonal variations of malaria transmission, particularly at low and medium intensity levels.

**Conclusion:**

The proposed definition discriminated well between studies with 'marked seasonality' and those with less seasonality. However, a poor fit was observed in sites with two seasonal peaks. Further work is needed to explore the applicability of this definition on a wide-scale, using routine health information system data where possible, to aid appropriate targeting of interventions.

## Background

Malaria remains the world's most important tropical parasitic disease, and one of the major public health challenges in the poorest countries of the world, particularly in sub-Saharan Africa. With the new move towards malaria eradication[1] and the scaling-up of malaria control interventions, there is a renewed energy and drive to maximize the impact of control tools in each epidemiological context. Where malaria transmission is seasonal, optimal timing of control becomes particularly important.

Knowing the duration, start and end of the malaria transmission season is important in terms of planning control strategies. For example, to maximize impact, Indoor Residual Spraying (IRS) should be carried out prior to the onset of the malaria season. Also, the cost-effectiveness of some malaria control strategies may be influenced by the intensity of seasonality. For example, in a setting where transmission only occurs during four months of the year, giving intermittent preventive treatment (IPT) to pregnant women (IPTp) or infants (IPTi) all year round would be less cost-effective than in a perennial transmission setting.

Most malaria endemic settings have "seasonal peaks" of malaria cases, which are usually described in terms of the duration and timing of the rains during a given study period. However, this may vary from year-to-year, giving a variety of subjective descriptions of seasonality for a single site. For example, in Manhica (Mozambique), malaria has been described as being perennial with "some" [2], "substantial"[3], and "marked"[4] seasonality, all of which are difficult to quantify. The challenge is how to translate this variation into a quantitative profile of malaria seasonality for a given area. This was highlighted by the need to undertake a pooled analysis of epidemiological data obtained from a systematic review, where it was necessary to define seasonality into distinct categories (Carneiro *et al*, submitted).

To date, several attempts have been made to describe the seasonality of malaria endemic areas such as the Climate and Malaria Resource Room (CMRR) [5] and Mapping Malaria Risk for Africa (MARA) collaboration [6]. The CMRR provides an interactive map that displays the number of months during the year when climatological conditions are considered to be suitable for malaria transmission. Although these maps provide a macro-scale illustration of where and when climatically-suitable conditions for malaria transmission exist, these continental maps do not account for micro-climatic conditions that can potentially have a significant role on actual transmission at a local scale[7]. The MARA collaboration, has developed maps that describe the expected duration and timing of the transmission season by country based on climate suitability models[8,9]. Although an attempt was made to validate these seasonal maps against parasite prevalence data, the statistical correlation between parasite prevalence and the number of months at malaria risk was found to be poor[6]. Furthermore, several authors have reported that the use of parasite prevalence data is not ideal for describing malaria seasonality [10-12], because at very high transmission levels malaria prevalence does not vary by season [13].

More recently, a different approach to define seasonality was carried out by Mabaso and others who aimed to predict seasonality from environmental covariates. They defined a seasonality concentration index to model the relationship between environmental covariates and seasonality in malaria incidence[14] and EIR data [12]. The authors reported that sites tended to show stronger seasonality of clinical malaria in all-year round transmission settings than in areas with shorter duration of malaria transmission, but no investigation was made on variations between years to look at consistency of findings[14]. To develop robust definitions, several years of data from each place are needed as there are annual variations, both in rainy seasons and in the intensity and timing of peaks in malaria.

Given that the intensity and seasonality of malaria transmission varies widely within and between countries in sub-Saharan Africa, the choice and timing of malaria control interventions might be different across distinct epidemiological contexts. This paper aims to present a simple method for describing the seasonality of malaria disease in a given site, to assist policy makers in deciding when and where malaria interventions should be delivered.

## Methods

### Data sources

**Figure 1:**
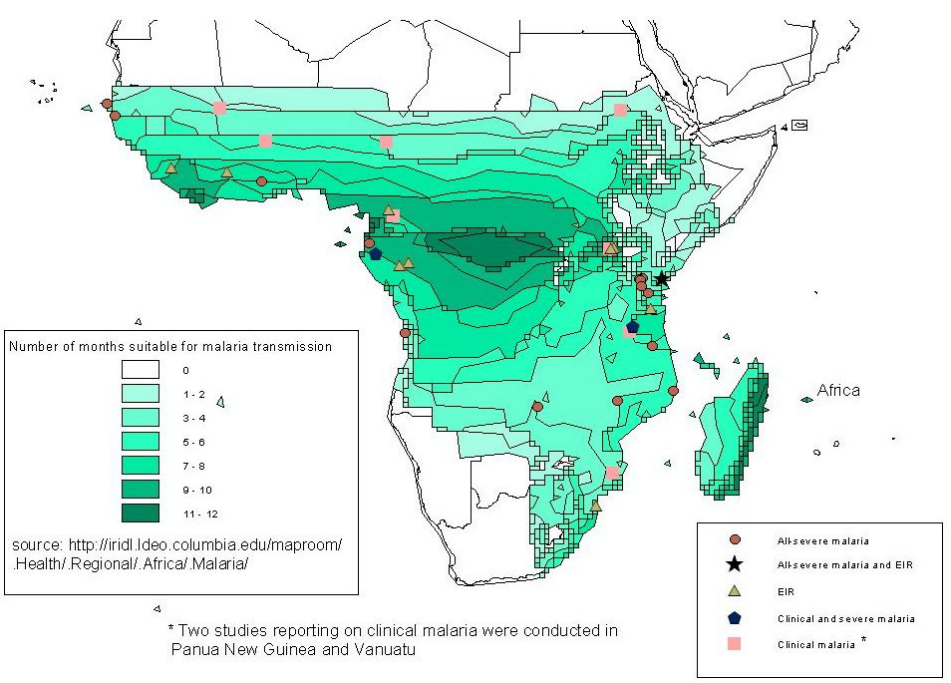
**Geographic location of sites reporting malaria data on full-academic years**.

A series of systematic literature reviews were undertaken using the PubMed and CAB Abstracts (BIDS) online abstracting databases, the WHO publication Library [15], and the SIGLE grey literature database[16] In October 2005, a review of studies measuring malaria morbidity outcomes between 1980-2005 was undertaken using the following search terms: malaria, falciparum, morbidity, mortality, epidemiology. In November 2005, a review of studies measuring entomological inoculation rates (EIR) between 1970-2005 was undertaken using combinations of the following search terms: EIR, entomologic* inoculation rate, sporozoites inoculation rate, anoph*, vector* capacity, biting rate, sporozoite rate, sporozoites index, malaria transmission, entomol*, malaria control, light trap, pyreth* spray, human bait. Only studies from sub-Saharan Africa and selected Pacific Ocean countries (Papua New Guinea, Solomon Islands, Vanuatu) endemic for *Plasmodium falciparum *and reporting on clinical malaria from community-based studies, hospital admissions with malaria, or entomological inoculation rates (EIR) for each month during a full calendar year were included (see Figure 1 and Table 1). Where additional information was needed, authors were contacted for further details. A number of authors shared individual-level datasets, or a more detailed monthly-breakdown of malaria outcome data than was publically available. Published and unpublished monthly data on EIR and malaria disease outcomes were compiled. Hospital admissions with malaria were broken-down where possible into the main recognized syndromes of severe malaria: cerebral malaria, severe malaria anaemia and respiratory distress, to ascertain whether patterns of seasonality varied by syndrome presentation.

**Table 1:** Study characteristics of sites included in the analyses

Country	Site	Seasonality	Reference	Study Period	Outcome data
Angola	Luanda	No marked	[§]	Jan to Dec 01	All-severe malaria

Cameroon	Ebolakounou	Marked	[31]	Apr 97 to Mar 98	Clinical malaria
	Simbok	Marked	[19]	Dec 98 to Nov 99	EIR

Cote d'Ivoire	Alloukoukro	Marked	[18]	Jan to Dec 91, Jan to Dec 92	EIR
			[18]		

Gabon	Akou	No marked	[25]	Jul 93 to Jun 94, Jul 94 to Jun 95	EIR
			[25]		
	Benguia	No marked	[25]	Aug 93 to Jul 94, Aug 94 to Jul 95, May 98 to Apr 99	EIR
		Marked	[25]		
		No marked	[25]		
	Dienga	Marked	[26]	May 95 to Apr 96	EIR
	Lambarene	No marked	[*]	Apr 03 to Mar 05	Clinical malaria
		No marked	[43]	Jan 01 to Dec 04	All-severe malaria
	Libreville	No marked	[43]	Feb 01 to Jan 05	All-severe malaria

Gambia, The	Banjul	Marked	[49]	Jan to Dec 91	All-severe malaria
		Marked	[#]	Jan 97 to Dec 01	All-severe malaria
		Marked	[43]	Apr 02 to Mar 05	All-severe malaria

Ghana	Kumasi	Borderline	[43]	Feb 01 to Jan 05	All-severe malaria
	Navrongo	Marked	[32]	Oct 00 to Sep 03	Clinical malaria

Kenya	Asembo Bay	No marked	[27]	Jan 03 to Dec 05	Clinical malaria
	Kilifi	No marked	[45]	Jan to Dec 91	All-severe malaria
		No marked	[43]	Jul 01 to Jun 05	All-severe malaria
	Kilifi District (9 villages)	Marked	[21]	Jun 92 to May 93	EIR
	Kisian	Borderline	[22]	Jan to Dec 86	EIR
	Saradidi	No marked	[22]	Jan to Dec 86	EIR

Mali	Kalanampala	Marked	[39]	Jan to Dec 97	Clinical malaria
	Tenegue	Marked	[39]	Jan to Dec 98	Clinical malaria

Malawi	Blantyre	Borderline	[43]	Apr 01 to Mar 05	All-severe malaria

Mozambique	Manhica	No marked	[2]	Jan 97 to Dec 97	Clinical malaria
		Borderline	[2]	Jan 98 to Dec 98	Clinical malaria
		No marked	[33]	Oct 02 to Sep 03	Clinical malaria
	Maputo	Marked	[42]	May 95 to Apr 97	All-severe malaria
	Matola/Maputo	No marked	[20]	Jan to Dec 95	EIR

Nigeria	Maiduguri	Marked	[47]	Jan 95 to Dec 96	All-severe malaria

PNG	Madang	No marked	[30]	Sep 90 to Aug 91	Clinical malaria

Senegal	Dakar	No marked	[45]	Oct 91 to Sep 92	All-severe malaria

Sierra Leone	Bo District	Marked	[23]	Jan to Dec 90	EIR

Eastern Sudan		No marked	[38]	Jan to Dec 97	Clinical malaria

Tanzania	Bagamoyo	Marked	[24]	Jan to Dec 90	EIR
	Huruma	Marked	[44]	Feb 02 to Jan 03	All-severe malaria
	Idete	No marked	[34,35,68]	Aug 93 to Jul 94	Clinical malaria
	Ifakara	No marked	[29]	Aug 99 to Jul 01	Clinical malaria
		Borderline	[28]	Jul 00 to Jun 01	Clinical malaria
		No marked	[41]	Jan to Dec 1995/2000	All-severe malaria
	Kibosho	No marked	[44]	Feb 02 to Jan 03	All-severe malaria
	Mnero	No marked	[44]	Jan 79 to Dec 81	All-severe malaria
	Moshi	No marked	[44]	Feb 02 to Jan 03	All-severe malaria
	Same	Borderline	[44]	Feb 02 to Jan 03	All-severe malaria
	Teule	No marked	[44]	Feb 02 to Jan 03	All-severe malaria

Uganda	Kampala	No marked	[37]	Nov 04 to Oct 05	Clinical malaria

Vanuatu		No marked	[40]	Jan 87 to Dec 90	Clinical malaria

Zambia	Macha	Marked	[**]	Jan 03 to Dec 04	All-severe malaria

§Bernardino L: Severe Malaria admissions in 2001. Luanda (Angola). Unpublished 2004

#Jallow M: Hospital Admissions with Severe Malaria Morbidity in RVTH, Banjul 1996-2002. MalariaGEN project, unpublished 2004.

*IPTi-Consortium: Lambarene (Gabon) IPTi trial, unpublished 2006.

**Thuma PE: Hospital Admissions of Severe Malaria Morbidity in Macha 2003-2004, unpublished 2004.

### Defining seasonality

Studies were only included if they had at least 50 cases of clinical or severe disease and monthly data for a full calendar year. To derive a definition of malaria seasonality, it was assumed that in areas with no seasonal peaks, *on average *50% of malaria episodes would be expected to occur within any given 6 months of the year. Conversely, in areas with seasonal peaks much more than 50% of malaria cases would be concentrated during the six-month period that includes the seasonal peak. To identify where this occurred, the percentage of cases occurring in each month of the year was calculated for each study. The cumulative percentage of all hospital admissions with malaria, clinical malaria incidence, EIR data and the number of months in which they happened were used to explore different definitions of 'marked seasonality'. Studies were defined as having 'marked seasonality' if 75% or more of the episodes occurred in 6 or less months of the year (definition A). Sites were defined as showing 'borderline marked seasonality' if 75% or more of the episodes occurred in seven months of the year.

The cumulative percentage was calculated for each consecutive six-month period in turn, using a rolling starting month, e.g. from January to June, then February to July, then March to August, and so-on up to December to May. The six-month combination that gave the highest cumulative percentage was defined as a "*concentrated period of malaria"*. Although age ranges varied by study, the analyses were conducted separately for each site and were, therefore, internally consistent.

Given the variability of EIR data collection methods, studies were screened for quality inclusion criteria based on methods of mosquito collection, frequency of sampling, method of sporozoite collection, and number of mosquitoes caught, tested and sporozoite positive. This led to the exclusion of one study where the methods were not fully described [13]. Furthermore, studies carried out in areas of low transmission intensity (annual EIR < 10 infective bites per person per year [ibpppy]) were also excluded[17] as they were considered to provide unreliable monthly EIR estimates. As the reliability of the seasonality patterns are likely to be dependent on the transmission intensity, studies are shown separately for EIRs of 10-100 ibpppy and > 100 ibpppy.

Sites that reported monthly data for a variety of outcomes were compared to see whether they showed a consistent definition of seasonality across outcomes. Further consistency checks included a comparison of the first and last month of the concentrated period of malaria (for sites found to show 'marked seasonality') with the first and last month of the rainy season (as reported in the literature) and with the first and last month of the malaria season based on the MARA predictions. Finally, to examine the robustness of our 'marked seasonality' definition, a sensitivity analysis was performed to test a variety of cut-offs against the original definition "A" (definition B: ≥ 75% of episodes in ≤ 3 months, definition C: ≥ 75% of episodes in ≤ 5 months, definition D: ≥ 75% of episodes in ≤ 7 months, definition E: ≥ 75% of episodes in ≤ 9 months, definition F: ≥ 70% of episodes in ≤ 6 months, and definition G: ≥ 80% of episodes in ≤ 6 months).

## Results

### Entomological inoculation rates (EIR)

**Figure 2:**
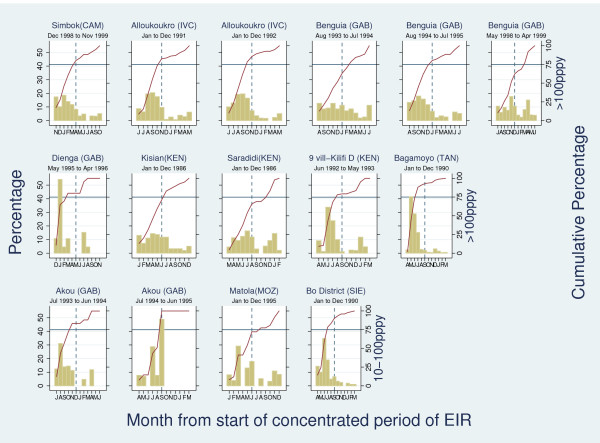
**Percentage (left-hand axis) and cumulative percentage (right-hand axis) of EIR by month from start of "concentrated period" (defined as 6 month period with maximal cumulative proportion of cases)**. The point at which the solid red line crosses the horizontal blue line defines the number of months in which 75% of cases occur. The blue dashed line crosses the horizontal blue line at month 6 of "concentrated period of cases". The first two rows refer to studies of annual EIR > 100 ibpppy. The last row refers to studies of 10-100 ibpppy annual EIR.

Annualized EIR data broken down by month were available from 11 sites[13,17-26]. For most sites with an annual EIR between 10-100 ibpppy (see Figure 2), the average number of infectious bites per person was null for some (non-consecutive) months of the year making it difficult to interpret the 'concentrated periods of malaria'. Only Bo District (Sierra Leone) was found to clearly show 'marked seasonality' in this transmission intensity category. In areas of annual EIR > 100 ibpppy, 'marked seasonality' was found in Simbok (Cameroon), Alloukoukro (Cote d'Ivoire), Dienga (Gabon), Kilifi (Kenya), and Bagamoyo (Tanzania). 'Borderline marked seasonality' was seen Kisian (Kenya). Benguia (Gabon) showed 'marked seasonality' in one out of three studies, suggesting that these differences might be due to annual variations (see Figure 2). More regular transmission with minor seasonal peaks was observed in the remaining sites.

### Clinical malaria

**Figure 3:**
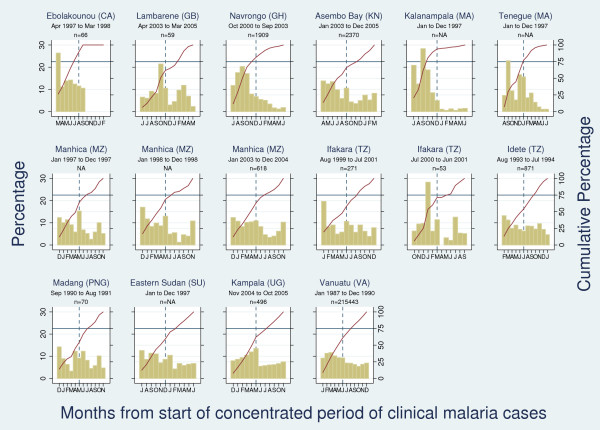
**Percentage (left-hand axis) and cumulative percentage (right-hand axis) of clinical malaria episodes among children by month from start of "concentrated period of cases" (defined as 6 month period with maximal cumulative proportion of cases)**. The point at which the solid red line crosses the horizontal blue line defines the number of months in which 75% of cases occur. The blue dashed line crosses the horizontal blue line at month 6 of "concentrated period of cases".

Individual-level data from nine sites with longitudinal data on more than 50 clinical malaria episodes were available for analysis [27-37]. In addition, monthly aggregated data were abstracted from four papers (five studies)[2,38-40]. Figure 3 shows that all sites exhibited year-round transmission except Ebolakounou (Cameroon), where clinical malaria cases were observed for only seven months of the year. 'Marked seasonality' (defined as more than 75% of all clinical malaria cases occurring in six or less months) was observed in Ebolakounou (Cameroon), Kalanampala (Mali), and Navrongo (Ghana), and Tenegue (Mali). Borderline seasonality was found in Manhica (Mozambique) and one of the two studies from Ifakara (Tanzania). More regular transmission with minor seasonal peaks was found in the other settings.

### All hospital admissions with malaria

Eighteen hospital-based sites across sub-Saharan Africa (20 studies) with data from full calendar years on hospital admissions with severe malaria were available for analysis (14 studies from individual-level datasets [41-44] [Jallow M: Hospital Admissions with Severe Malaria Morbidity in RVTH, Banjul 1996-2002, MalariaGEN project, 2004 unpublished and Thuma PE: Hospital Admissions of Severe Malaria Morbidity in Macha 2003-2004, 2004 unpublished] and six studies obtained from monthly aggregated data [45-49] [Bernardino L: Severe Malaria admissions in 2001. Luanda (Angola), unpublished 2004].

**Figure 4:**
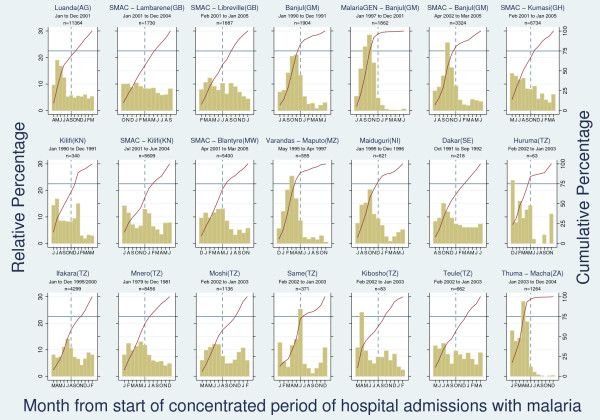
**Percentage (left-hand axis) and cumulative percentage (right-hand axis) of hospital admissions with malaria among children by month from start of "concentrated period of cases" (defined as 6 month period with maximal cumulative proportion of cases)**. The point at which the solid red line crosses the horizontal blue line defines the number of months in which 75% of cases occur. The blue dashed line crosses the horizontal blue line at month 6 of "concentrated period of cases".

Perennial transmission with 'marked seasonality' was found in Banjul (The Gambia), Maputo (Mozambique), Macha (Zambia), Maiduguri (Nigeria), and Huruma (Tanzania). 'Borderline marked seasonality' was seen in Kumasi (Ghana), Blantyre (Malawi), and Same (Tanzania). More regular transmission with minor seasonal peaks was found in the remaining sites (see Figure 4).

### Consistency of seasonality results across sites

**Figure 5:**
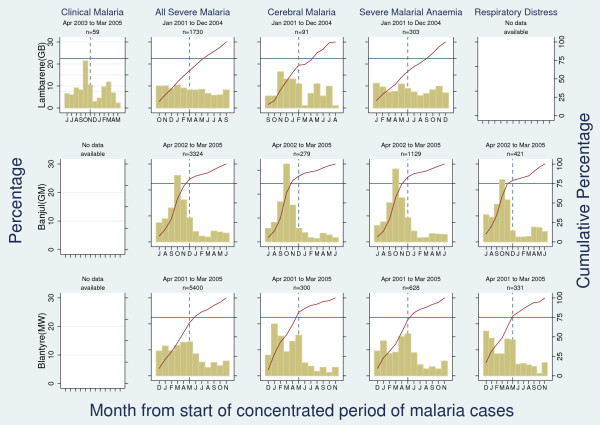
**Three examples of consistency of seasonality findings across outcomes: Lambarene (Gabon) as "no marked seasonality" (top row), Banjul (The Gambia) as "marked seasonality" (middle row), and Blantyre (Malawi) as "borderline marked seasonality" (bottom row)**.

**Figure 6:**
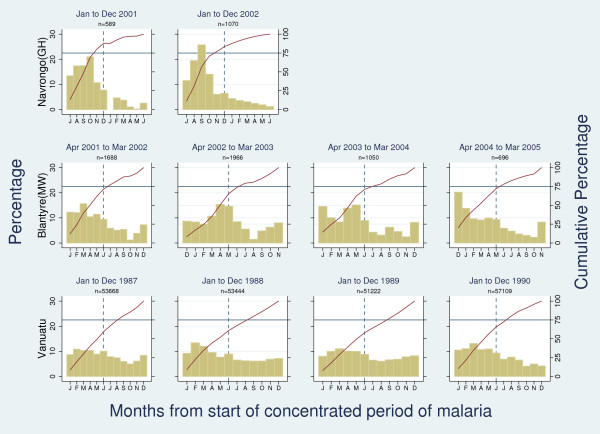
**Three examples of consistency of seasonality findings across calendar year: Navrongo (Ghana) as "marked seasonality" (top row), Blantyre (Malawi) as "borderline marked seasonality" (middle row), and Vanuatu as "no marked seasonality" (bottom row)**.

Overall, 15 sites were found to present 'marked seasonality' (see additional file 1). Of these, more than one malaria outcome was available for Banjul (The Gambia), Macha (Zambia), Maputo (Mozambique), and Kilifi (Kenya). With regards to malaria morbidity outcomes, a consistent 'concentrated period of malaria' was found in all sites showing 'marked seasonality'. In Banjul, for example, the 'concentrated period of malaria' was consistently found to run from July to December when looking at all hospital admissions with malaria, cerebral malaria, severe malarial anaemia, and respiratory distress respectively. Consistent results were also observed for sites not meeting our 'marked seasonality' definition (see Figure 5 for three examples). When data were available from more than one calendar year (15 sites) similar seasonality patterns were seen within each setting (see Figure 6 for three examples of 'no marked seasonality', 'marked seasonality', and 'borderline marked seasonality').

For sites reporting on both EIR and malaria morbidity outcomes (Maputo and Kilifi), results showed some discrepancies as not all outcomes consistently fitted our 'marked seasonality' definition. In Maputo, more than 75% of all hospital admissions with malaria were found to be concentrated between December and May, whereas EIR data did not meet the proposed 'marked seasonality' definition. Conversely, Kilifi (Kenya) showed 'marked seasonality' when EIR data were analysed, but 'no marked seasonality' in the two studies reporting on all hospital admissions with malaria. These results suggest that EIR data are less reliable for assessing malaria seasonality than malaria morbidity outcomes.

### Validation of concentrated periods of malaria

The range of months of the concentrated period of malaria for all sites that showed 'marked seasonality' of clinical malaria and of hospital admissions with malaria were found to be concordant and fully overlapped with the duration and timing of the rainy season as reported in the literature and/or the first/last month of seasonality as estimated by MARA climate suitability models (see Additional file 1). Some of the sites found to have 'poor agreement' (if the estimated 'concentrated period of malaria' did not fully overlap with the author's definitions or the MARA maps) could be explained by the fact that 'concentrated periods of malaria' tended to start one or two months after the start of the rainy season, which is likely to be due to the time lag between onset of rains, increase in mosquito density and presentation of symptoms. For example, rains in Navrongo (Ghana), and Kalanampala (Mali) were reported to usually occur between June and October, whereas, the 'concentrated period of malaria' was found to occur from July to December. A poor agreement was also observed for sites experiencing two rainy seasons suggesting that our method is less reliable for defining seasonal patterns of malaria transmission in these settings.

### Sensitivity analysis

To examine the robustness of our definition of 'marked seasonality', several sensitivity analyses were performed based on a variety of cut-offs (see Additional file 2).

All sites found to have 'marked seasonality' according to the original definition A of ≥ 75% of episodes in ≤ 6 months, also showed 'marked seasonality' when the period was restricted to five months (definition B). As expected, when the cut-off for cumulative proportion of cases was reduced to ≥ 70% of episodes in ≤ 6 months (definition C) a few additional sites were found to fulfil the criteria for 'marked seasonality' (Ifakara, Blantyre, and Kumasi). Conversely, when the cut-off for cumulative proportion of cases was increased to ≥ 80% (definition D), Blantyre was no longer considered to show 'borderline marked seasonality'. These results suggest that a consecutive period of 5-6 months gives a consistent seasonality pattern.

## Discussion

In this paper, a standardized and practical approach for defining seasonality of malaria is presented based on a simple statistical analysis of the monthly distribution of malaria disease from a wide range of epidemiological settings across malaria endemic countries. A proposed definition of 'marked seasonality', where 75% or more of the episodes occurred in six or less months of the year, appeared to discriminate well between studies with 'marked seasonality' of cases and those with a more regular distribution of cases and perhaps one or two seasonal peaks. Furthermore, consistent results were observed when more than one outcome or more than one calendar year was available from the same site. With regards to monthly EIR data, however, a reliable assessment of seasonality could only be made in sites with high transmission (i.e. > 100 ibpppy), where monthly EIR data was greater than 0 for most consecutive months of the year. The 'concentrated period of malaria' in less intense transmission settings is difficult to interpret as it is not clear whether there is actually no transmission during those months of the year or whether monthly EIR measurements in these areas suffer from poor sensitivity (as monthly sporozoite rates tend to be very low or null yielding zero EIR values during times of the year when there may actually be malaria transmission[50]). Furthermore, for a given site, results from EIR data were not fully consistent with those of malaria morbidity outcomes suggesting that the use of monthly EIR data is limited when looking at seasonal variations of malaria transmission.

For example, Kilifi (Kenya) was found to show 'borderline seasonality' when monthly EIR data was analysed, but 'no marked seasonality' in the two sites reporting data on all hospital admissions with malaria. This discrepancy might also be due to the fact that Kilifi is usually reported as having two rainy seasons[17,51-55]. The proposed definition of 'marked seasonality' might not be appropriate for sites with strongly bimodal seasonal patterns, as the highest proportion of cases may not occur in consecutive months. Difficulty in defining malaria seasonality in these areas has also been reported in previous attempts[12]. Further work is, therefore, needed to provide a quantitative description of malaria seasonality in these settings.

The range of months of the concentrated period of malaria for sites showing 'marked seasonality', were found to be concordant with both the rainy periods reported in the literature and first/last month of the MARA seasonality database indicating that our approach works well for identifying these aspects of seasonality in these study settings (see Additional file 1). Again, the few discrepancies found between the concentrated period of malaria and the rainy season corresponded to those sites reporting two peaks of rain, corroborating the poor fit of our method in identifying the concentrated period of malaria in these settings. A better fit was usually observed between the concentrated period of malaria and the rainy season in sites where the source of information on the rainy season was the paper that reported the monthly malaria data. This is likely to be due to year-to-year variation in the onset of the rainy season, resulting in a better fit when the onset of the rainy season is matched to the period of clinical surveillance. As can be seen in Additional file 1, the concentrated period of malaria tended to start one month after the start of the rainy season. As it usually takes a few weeks from the start of the rainy season for a sufficient increase in mosquito abundance to reflect a substantial increase of malaria cases[26,56], it is therefore crucial to take this into consideration to maximize impact of malaria control interventions. For example, IRS interventions should be carried out at least one month prior to the expected onset of the identified concentrated period of malaria in a given area.

The proposed method for identifying the start and end of a concentrated period of malaria overcomes some of the limitations of using the low-spatial resolution seasonality MARA maps to identify the start and end of the malaria season at a local scale which usually results in errors particularly in areas where environmental conditions are most variable[57,58].

A common definition of malaria seasonality is also crucial for the comparability of studies. The proposed method has already been applied to describe the age-pattern of malaria of differing severities across a range of transmission settings on a series of pooled analyses of existing data (Carneiro *et al*, submitted and Roca-Feltrer *et al*, manuscript in preparation). The use of a standardized definition of malaria seasonality in these comparative studies has improved our ability to discriminate studies with 'marked seasonality' from those with more regular transmission and seasonal peaks.

With the rapid scaling up of malaria control interventions in sub-Saharan African countries, continued surveillance is needed to monitor changes in transmission intensity levels and in the burden of malaria disease. Several authors have already reported a drop in hospital malaria admissions after analysing several years of surveillance data [50,59-61]. Although there may be several reasons for the observed declines in malaria morbidity, authors have argued that decreases in transmission intensity and/or increases in malaria intervention coverage are likely to have played an important role in the changing pattern of malaria epidemiology in these settings. But does malaria seasonality change over time as transmission levels decrease in a given setting? In an area of marked seasonality in The Gambia, for example, the same seasonal peaks were observed every year between 2001 and 2007[50] suggesting that the proposed method for defining a concentrated period of malaria may remain useful even when transmission intensity decreases over time. Further work is needed however to assess whether changes in seasonality may occur with declining transmission intensity in areas of perennial transmission.

Although results from these analyses are encouraging for defining malaria seasonality, in practice there is little reliable monthly data on clinical and severe malaria in many parts of sub-Saharan Africa, questioning its utility on a wide scale. Health Management Information Systems (HMIS) -when working well-have been shown to provide consistent seasonal variation in the number of cases seen at outpatient clinics, coinciding with the pattern of malaria transmission [62]. Although HMIS are often unreliable, sentinel sites -with usually improved quality of data collection-can be used to further explore the applicability of this method. This could help to inform policy makers at a local level to optimize the timing and frequency of indoor residual spraying with insecticides or campaigns to promote the use of insecticide-treated bed nets, as is being done with epidemic early warning systems [63,64]. Application of this approach to routinely collected local data could also be beneficial in targeting seasonally focussed malaria interventions, such as the seasonal administration of intermittent preventive treatment to children (IPTc) [65-67].

## Conclusion

Currently, there is no standard way of defining malaria seasonality resulting in a wide range of definitions reported in the literature. Where there is considerable seasonality, optimal timing of control becomes particularly important. Here a simple approach for describing the seasonality of malaria is presented, that discriminates well between studies with 'marked seasonality' (75% or more of malaria episodes occurring in six or less months of the year) and those with less seasonality. This method also helps to identify the start and end of a 'concentrated period of malaria' at a local scale, which could inform policy makers about the optimum timing and frequency of deployment of malaria control interventions. A poor fit, however, was observed in sites with two seasonal peaks. Further work is needed to explore the applicability of this method, using routine health information system data.

## Abbreviations

EIR: Entomological Inoculation Rate; Ibpppy: infective bites per person per year; IPT: intermittent preventive treatment; IRS: indoor residual spraying; MARA: Mapping Malaria Risk for Africa.

## Competing interests

The authors declare that they have no competing interests.

## Authors' contributions

ARF, JAS & IC conceived and designed the analyses, ARF & LS collected the data, ARF analysed the data. All authors contributed to the writing of the manuscript and approved the final version.

## Supplementary Material

Additional file 1 **Comparison of malaria concentrated periods (i.e. month interval in which 75% of cases occurred) with the month interval of the rainy season as defined by the literature and/or by MARA for sites found to show 'marked seasonality'.** Sites were defined as having 'poor agreement' if the 'concentrated period of malaria' did not fully overlapped with either the reported rainy months or the MARA maps. Conversely, sites were defined as having 'good agreement' if the 'concentrated period of malaria' fully overlapped with at least one of them. Click here for file

Additional file 2 **Table summarising results from sensitivity analyses looking at different season lengths (3, 5, 7, and 9 months) and different cumulative proportion cut-offs.** Cumulative percentages for definition A (proposed definition for identifying sites showing 'marked seasonality') is also shown for comparison purposes. Click here for file
