# Supplementary material for: A simple method for defining malaria seasonality
Source: Malar J. 2009 Dec 3;8:276. doi: 10.1186/1475-2875-8-276 (PMC3224898; doi:10.1186/1475-2875-8-276)
Supplement: Additional file 1 — Comparison of malaria concentrated periods (i.e. month interval in which 75% of cases occurred) with the month interval of the rainy season as defined by the literature and/or by MARA for sites found to show 'marked seasonality'. Sites were defined as having 'poor agreement' if the 'concentrated period of malaria' did not fully overlapped with either the reported rainy months or the MARA maps. Conversely, sites were defined as having 'good agreement' if the 'concentrated period of malaria' fully overlapped with at least one of them. [file 1475-2875-8-276-S1.DOCX]

**Additional file 1** - Comparison of malaria concentrated periods (i.e. month interval in which 75% of cases occurred) with the month interval of the rainy season as defined by the literature and/or by MARA for sites found to show ‘marked seasonality’. Sites were defined as having ‘poor agreement’ if the ‘concentrated period of malaria’ did not fully overlapped with either the reported rainy months or the MARA maps. Conversely, sites were defined as having ‘good agreement’ if the ‘concentrated period of malaria’ fully overlapped with at least one of them.

| **Site (Country)** | **Months of concentrated period of malaria cases** | | | | | | **Months of rainy season^¥^** | **Months of MARA malaria season** | **Level of agreement^ŧ^** |
| --- | --- | --- | --- | --- | --- | --- | --- | --- | --- |
|  | **Clinical Malaria** | **Hospital admissions with malaria** | **CM** | **SMA** | **RD** | **EIR** |  |  |  |
| Ebolakounou (Cameroon) | Mar-Aug | - | - | - | - | - | **Short: Apr-May Long: Aug-Nov**[1] | Mar-Dec | Good |
| Simbok (Cameroon) | - | - | - | - | - | Nov-Apr | **Short: Sep-Nov Long: Mar-Jun**[2] | Mar-Dec | Poor |
| Alloukoukro  (Cote d'Ivoire)^§^ | - | - | - | - | - | Jun-Nov | **Mar-Oct**[3] | Apr-Dec | Good |
| Benguia (Gabon)^§^ | - | - | - | - | - | Apr-Jun | **Short: Oct-Dec Long: Mar-Jun** [4] | Oct-Aug | Good |
| Dienga (Gabon) | - | - | - | - | - | Dec-May | **Short: Sep-Dec Long: Mar-Jun**  [5] | Oct-Aug | Good |
| Navrongo (Ghana) | Jul-Dec | - | - | - | - | - | **Jun-Oct**[6] | Jun-Nov | Poor |
| Kilifi (Kenya) | - | - ^Ω^ | - | - | - | Apr-Sep | Short: Oct-Nov Long: Apr-Jun[7-9]  Short: Sep-Oct Long: Apr-Jul[10]  Short: Oct-Dec Long: Apr-Aug[11] | Apr-Dec | Good |
| Kalanampala (Mali) | Jul-Dec | - | - | - | - | - | **Jun-Oct**[12] | Jul-Oct | Poor |
| Tenegue (Mali) | Aug-Jan | - | - | - | - | - | **Jun-Oct**[12] | Jul-Oct | Poor |
| Maputo (Mozambique) | - | Dec-May | Dec-May | Dec-May | Dec-May | -^Ω^ | Oct-May[13]  **Nov-Apr**[14] | Dec-Apr | Good |
| Bo District (Sierra Leone) | - | - | - | - | - | Apr-Sep | **May-Nov**[15]  May-Oct[16, 17] | Apr-Jan | Good |
| Bagamoyo (Tanzania) | - | - | - | - | - | May-Oct | **Short: Nov-Dec Long: Mar-Jul**[18] | Dec-Jul | Poor |
| Huruma (Tanzania) | - | Dec-May | - | - | - | - | **Short: Nov-Dec Long: Mar-May**[19] | Nov-May | Good |
| RVTH  (The Gambia)^∫^ | - | Jul-Dec | Jul-Dec | Jul-Dec | Jul-Dec | - | Jun-Oct[20]  **Jul-Oct**[21-24]  Jul-Nov[25, 26]  Jul-Dec[27]  **Aug-Dec**[28] | Jul-Nov | Good |
| Macha (Zambia) | - | Jan-Jun | Jan-Jun | Jan-Jun | - | - | Nov-Apr[29, 30] | Dec-Feb | Poor |

^¥^Periods shown in bold refer to rainy seasons as reported by papers from which we obtained monthly data

^∫^ Same results obtained for [21, 27, 28, 31]

^§^ Same results obtained in 1991 and 1992 for Alloukoukro and in 1993 and 1994 for Benguia

^Ω^Data available for this outcome but site not found to show ‘marked seasonality’

**References**

1. Bonnet S, Paul RE, Gouagna C, Safeukui I, Meunier JY, Gounoue R, Boudin C: **Level and dynamics of malaria transmission and morbidity in an equatorial area of South Cameroon**. *Trop Med Int Health* 2002, **7**:249-256.

2. Antonio-Nkondjio C, Awono-Ambene P, Toto JC, Meunier JY, Zebaze-Kemleu S, Nyambam R, Wondji CS, Tchuinkam T, Fontenill D: **High malaria transmission intensity in a village close to Yaounde, the capital city of Cameroon**. *J Med Entomol* 2002, **39**:350-355.

3. Dossou-yovo J, Doannio JM, Riviere F, Chauvancy G: **Malaria in Cote d'Ivoire wet savannah region: the entomological input**. *Trop Med Parasitol* 1995, **46**:263-269.

4. Elissa N, Karch S, Bureau P, Ollomo B, Lawoko M, Yangari P, Ebang B, Georges AJ: **Malaria transmission in a region of savanna-forest mosaic, Haut-Ogooue, Gabon**. *J Am Mosq Control Assoc* 1999, **15**:15-23.

5. Elissa N, Migot-Nabias F, Luty A, Renaut A, Toure F, Vaillant M, Lawoko M, Yangari P, Mayombo J, Lekoulou F, Tshipamba P, Moukagni R, Millet P, Deloron P: **Relationship between entomological inoculation rate, *Plasmodium falciparum* prevalence rate, and incidence of malaria attack in rural Gabon**. *Acta Trop* 2003, **85**:355-361.

6. Chandramohan D, Owusu-Agyei S, Carneiro I, Awine T, Amponsa-Achiano K, Mensah N, Jaffar S, Baiden R, Hodgson A, Binka F, Greenwood B: **Cluster randomised trial of intermittent preventive treatment for malaria in infants in area of high, seasonal transmission in Ghana**. *BMJ* 2005, **331**:727-733.

7. Mbogo CN, Snow RW, Kabiru EW, Ouma JH, Githure JI, Marsh K, Beier JC: **Low-level *Plasmodium falciparum* transmission and the incidence of severe malaria infections on the Kenyan coast**. *Am J Trop Med Hyg* 1993, **49**:245-253.

8. Mbogo CM, Mwangangi JM, Nzovu J, Gu W, Yan G, Gunter JT, Swalm C, Keating J, Regens JL, Shililu JI, Githure JI, Beier JC: **Spatial and temporal heterogeneity of Anopheles mosquitoes and *Plasmodium falciparum* transmission along the Kenyan coast**. *Am J Trop Med Hyg* 2003, **68**:734-742.

9. O'Meara WP, Bejon P, Mwangi TW, Okiro EA, Peshu N, Snow RW, Newton CRJC, Marsh K: **Effect of a fall in malaria transmission on morbidity and mortality in Kilifi , Kenya**. *Lancet* 2008, **372**:1555–1562.

10. Snow RW, Bastos de Azevedo I, Lowe BS, Kabiru EW, Nevill CG, Mwankusye S, Kassiga G, Marsh K, Teuscher T: **Severe childhood malaria in two areas of markedly different falciparum transmission in east Africa**. *Acta Trop* 1994, **57**:289-300.

11. Snow RW, Omumbo JA, Lowe B, Molyneux CS, Obiero JO, Palmer A, Weber MW, Pinder M, Nahlen B, Obonyo C, Newbold C, Gupta S, Marsh K: **Relation between severe malaria morbidity in children and level of Plasmodium falciparum transmission in Africa**. *Lancet* 1997, **349**:1650-1654.

12. Sissoko MS, Dicko A, Briet OJ, Sissoko M, Sagara I, Keita HD, Sogoba M, Rogier C, Toure YT, Doumbo OK: **Malaria incidence in relation to rice cultivation in the irrigated Sahel of Mali**. *Acta Trop* 2004, **89**:161-170.

13. Romagosa C, Ordi J, Saute F, Quinto L, Machungo F, Ismail MR, Carrilho C, Osman N, Alonso PL, Menendez C: **Seasonal variations in maternal mortality in Maputo, Mozambique: the role of malaria**. *Trop Med Int Health* 2007, **12**:62-67.

14. Mendis C, Jacobsen JL, Gamage-Mendis A, Bule E, Dgedge M, Thompson R, Cuamba N, Barreto J, Begtrup K, Sinden RE, Hogh B: ***Anopheles arabiensis* and *An. funestus* are equally important vectors of malaria in Matola coastal suburb of Maputo, southern Mozambique**. *Med Vet Entomol* 2000, **14**:171-180.

15. Bockarie MJ, Service MW, Barnish G, Maude GH, Greenwood BM: **Malaria in a rural area of Sierra Leone. III. Vector ecology and disease transmission**. *Ann Trop Med Parasitol* 1994, **88**:251-262.

16. Magbity EB, Marbiah NT, Maude G, Curtis CF, Bradley DJ, Greenwood BM, Petersen E, Lines JD: **Effects of community-wide use of lambdacyhalothrin-impregnated bednets on malaria vectors in rural Sierra Leone**. *Med Vet Entomol* 1997, **11**:79-86.

17. Barnish G, Maude GH, Bockarie MJ, Erunkulu OA, Dumbuya MS, Greenwood BM: **Malaria in a rural area of Sierra Leone. II. Parasitological and related results from pre- and post-rains clinical surveys**. *Ann Trop Med Parasitol* 1993, **87**:137-148.

18. Shiff CJ, Minjas JN, Hall T, Hunt RH, Lyimo S, Davis JR: **Malaria infection potential of anopheline mosquitoes sampled by light trapping indoors in coastal Tanzanian villages**. *Med Vet Entomol* 1995, **9**:256-262.

19. Reyburn H: **Dry and Wet seasons for Northern Tanzania**. Personal communication (2007)

20. Bojang KA, Hensbroek MBv, Palmer A, Banya WAS, Jaffar S, Greenwood BM: **Predictors of mortality in Gambian children with severe malaria anaemia**. *Annals of Tropical Paediatrics* 1997, **17**:355-359.

21. Brewster DR, Kwiatkowski D, White NJ: **Neurological sequelae of cerebral malaria in children**. *Lancet* 1990, **336**:1039-1043.

22. Hensbroek MBv, Palmer A, Jaffar S, Schneider G, Kwiatkowski D: **Residual neurologic sequelae after childhood cerebral malaria**. *J Pediatr* 1997, **131**:125-129.

23. Alonso PL, Lindsay SW, Armstrong JR, Conteh M, Hill AG, David PH, Fegan G, de Francisco A, Hall AJ, Shenton FC, et al.: **The effect of insecticide-treated bed nets on mortality of Gambian children**. *Lancet* 1991, **337**:1499-1502.

24. Jaffar S, Leach A, Greenwood AM, Jepson A, Muller O, Ota MO, Bojang K, Obaro S, Greenwood BM: **Changes in the pattern of infant and childhood mortality in upper river division, The Gambia, from 1989 to 1993**. *Trop Med Int Health* 1997, **2**:28-37.

25. Snow RW, Rowan KM, Greenwood BM: **A trial of permethrin-treated bed nets in the prevention of malaria in Gambian children**. *Trans R Soc Trop Med Hyg* 1987, **81**:563-567.

26. Otoo LN, Snow RW, Menon A, Byass P, Greenwood BM: **Immunity to malaria in young Gambian children after a two-year period of chemoprophylaxis**. *Trans R Soc Trop Med Hyg* 1988, **82**:59-65.

27. Brewster DR, Greenwood BM: **Seasonal variation of paediatric diseases in The Gambia, West Africa**. *Ann Trop Paediatrics* 1993, **13**:133-146.

28. Taylor T, Olola C, Valim C, Agbenyega T, Kremsner P, Krishna S, Kwiatkowski D, Newton C, Missinou M, Pinder M, Wypij D: **Standardized data collection for multi-center clinical studies of severe malaria in African children: establishing the SMAC network**. *Trans R Soc Trop Med Hyg* 2006, **100**:615-622.

29. Kent RJ, Thuma PE, Mharakurwa S, Norris DE: **Seasonality, blood feeding behavior, and transmission of *Plasmodium falciparum* by *Anopheles arabiensis* after an extended drought in southern Zambia**. *Am J Trop Med Hyg* 2007, **76**:267-274.

30. Biemba G, Dolmans D, Thuma PE, Weiss G, Gordeuk VR: **Severe anaemia in Zambian children with Plasmodium falciparum malaria**. *Tropical Medicine and International Health* 2000, **5**:9-16.

31. Jallow M: **Hospital Admissions with Severe Malaria Morbidity in RVTH, Banjul 1996-2002. MalariaGEN project**. In*.*; Personal communication (2004).
