# Supplementary material for: A simple method for defining malaria seasonality
Source: Malar J. 2009 Dec 3;8:276. doi: 10.1186/1475-2875-8-276 (PMC3224898; doi:10.1186/1475-2875-8-276)
Supplement: Additional file 2 — Table summarising results from sensitivity analyses looking at different season lengths (3, 5, 7, and 9 months) and different cumulative proportion cut-offs. Cumulative percentages for definition A (proposed definition for identifying sites showing 'marked seasonality') is also shown for comparison purposes. [file 1475-2875-8-276-S2.DOC]

**Additional File 2.** Table summarising results from sensitivity analyses looking at different season lengths (3, 5, 7, and 9 months) and different cumulative proportion cut-offs. Cumulative percentages for definition A (proposed definition for identifying sites showing ‘marked seasonality’) is also shown for comparison purposes.

| **Site (country)** | **Reference** | **Highest cumulative proportion of cases (%) in** | | | | | | |
| --- | --- | --- | --- | --- | --- | --- | --- | --- |
|  |  | **3 consecutive months** | **5 consecutive months** | **6 consecutive months** | | | **7 consecutive months** | **9 consecutive months** |
| **Definition**^¥^ |  | **B** | **C** | **A** | **F** | **G** | **D** | **E** |
| **Clinical malaria** | | | | | | | | |
| Ebolakounou (Cameroon) | [1] | 51.3 | **77.9** | **89.5** | **89.5** | **89.5** | **100** | **100** |
| Lambarene (Gabon) | [2] | 40.2 | 55.3 | 61.9 | 61.9 | 61.9 | 69.1 | **85.2** |
| Navrongo (Ghana) | [3] | 55.0 | 74.7 | **81.5** | **81.5** | **81.5** | **86.9** | **95.4** |
| Asembo (Kenya) | [4] | 39.8 | 57.9 | 66.2 | 66.2 | 66.2 | 74.4 | **87.1** |
| Kalanampala (Mali) | [5] | 61.7 | **89.0** | **94.0** | **94.0** | **94.0** | **95.4** | **97.7** |
| Tenegue (Mali) | [5] | 39.6 | 69.8 | **77.1** | **77.1** | 77.1 | **84.4** | **95.9** |
| Manhica (Mozambique)^∫^ | [6] | 33.9 | 51.8 | 64.2 | 64.2 | 64.2 | 71.0 | **86.3** |
| Manhica (Mozambique)^∫^ | [6] | 39.6 | 51.7 | 68.7 | 68.7 | 68.7 | **79.6** | **90.1** |
| Manhica (Mozambique)^∫^ | [7] | 33.4 | 52.1 | 63.1 | 63.1 | 63.1 | 73.4 | **87.9** |
| Madang (PNG) | [8] | 33.8 | 46.5 | 54.3 | 54.3 | 54.3 | 66.6 | **81.5** |
| Eastern Sudan | [9] | 32.6 | 50.8 | 59.4 | 59.4 | 59.4 | 69.8 | **83.1** |
| Idete (Tanzania) | [10-12] | 32.1 | 48.1 | 54.4 | 54.4 | 54.4 | 63.0 | **78.7** |
| Ifakara (Tanzania) | [13] | 36.9 | 49.7 | 58.3 | 58.3 | 58.3 | 67.3 | **82.1** |
| Ifakara (Tanzania) | [14] | 47.5 | 65.2 | 71.5 | **71.5** | 71.5 | **77.0** | **94.6** |
| Kampala (Uganda) | [15] | 36.0 | 54.1 | 62.1 | 62.1 | 62.1 | 69.6 | **82.6** |
| Vanuatu | [16] | 33.2 | 51.9 | 61.1 | 61.1 | 61.1 | 68.0 | **81.8** |
| **Hospital admissions with malaria** | | | | | | | | |
| Luanda (Angola) | [17] | 48.2 | 63.1 | 68.7 | 68.7 | 68.7 | 74.0 | **84.5** |
| Lambarene (Gabon) | [18] | 29.3 | 48.4 | 56.7 | 56.7 | 56.7 | 64.9 | **81.6** |
| Libreville (Gabon) | [18] | 32.0 | 50.0 | 57.9 | 57.9 | 57.9 | 66.6 | **83.5** |
| Banjul (Gambia, The) | [19] | 55.7 | **76.7** | **83.7** | **83.7** | **83.7** | **89.0** | **94.4** |
| Banjul (Gambia, The) | [20] | 70.6 | **91.8** | **96.5** | **96.5** | **96.5** | **98.5** | **99.2** |
| Banjul (Gambia, The) | [18] | 54.9 | 73.1 | **80.5** | **80.5** | **80.5** | **84.5** | **91.9** |
| Kumasi (Ghana) | [18] | 38.6 | 59.0 | 67.0 | 67.0 | 67.0 | **76.2** | **87.0** |
| Kilifi (Kenya) | [21] | 39.7 | 56.4 | 64.6 | 64.6 | 64.6 | 74.6 | **91.9** |
| Kilifi (Kenya) | [18] | 33.5 | 48.3 | 58.0 | 58.0 | 58.0 | 69.3 | **84.8** |
| Blantyre (Malawi) | [18] | 37.6 | 58.8 | 68.4 | 68.4 | 68.4 | **76.9** | **87.9** |
| Maputo (Mozambique) | [22] | 62.5 | **83.2** | **88.8** | **88.8** | **88.8** | **91.1** | **95.0** |
| Maiduguri (Nigeria) | [23] | 50.1 | 70.4 | **78.0** | **78.0** | 78.0 | **84.8** | **94.2** |
| Dakar (Senegal) | [24] | 36.6 | 54.5 | 61.8 | 61.8 | 61.8 | 69.1 | **82.0** |
| Huruma (Tanzania) | [25] | 47.6 | 69.8 | **84.1** | **84.1** | **84.1** | **95.2** | **98.4** |
| Ifakara (Tanzania) | [26] | 35.8 | 50.4 | 58.3 | 58.3 | 58.3 | 66.4 | **82.5** |
| Kibosho (Tanzania) | [25] | 44.5 | 55.3 | 62.5 | 62.5 | 62.5 | 69.7 | **85.3** |
| Mnero (Tanzania) | [25] | 36.0 | 58.2 | 66.3 | 66.3 | 66.3 | 74.3 | **85.6** |
| Moshi (Tanzania) | [25] | 35.1 | 52.5 | 58.5 | 58.5 | 58.5 | 67.2 | **84.3** |
| Same (Tanzania) | [25] | 50.9 | 59.8 | 72.5 | **72.5** | 72.5 | **83.3** | **93.0** |
| Teule (Tanzania) | [25] | 34.3 | 51.5 | 56.8 | 56.8 | 56.8 | 65.7 | **80.4** |
| Macha (Zambia) | [27] | 63.2 | **93.6** | **97.2** | **97.2** | **97.2** | 98.8 | **99.7** |
| **Entomological Inoculation Rates** | | | | | | | | |
| Simbok (Cameroon) | [28] | 46.2 | 72.0 | **80.2** | **80.2** | **80.2** | **85.3** | **93.5** |
| Alloukoukro (Cote d’Ivoire) ^∫^ | [29] | 57.7 | 73.9 | **82.7** | **82.7** | **82.7** | **88.9** | **95.7** |
| Alloukoukro (Cote d’Ivoire) ^∫^ | [29] | 55.4 | **78.4** | **85.7** | **85.7** | **85.7** | **90.8** | **96.7** |
| Akou (Gabon)^∫^ | [30]  [30] | 59.6 | **83.3** | **83.3** | **83.3** | **83.3** | **83.9** | **95.2** |
| Akou (Gabon)^∫^ | [30]  [30]  [30] | 85.4 | **92.3** | **100** | **100** | **100** | **100** | **100** |
| Benguia (Gabon)^∫^ | [30] | 32.5 | 54.4 | 63.0 | 63.0 | 63.0 | 74.2 | **90.0** |
| Benguia (Gabon)^∫^ | [30]  [30] | 48.3 | 73.3 | **80.1** | **80.1** | **80.1** | **85.3** | **93.8** |
| Benguia (Gabon)^∫^ | [30]  [30]  [30] | 35.9 | 51.6 | 62.2 | 62.2 | 62.2 | 66.2 | **85.4** |
| Dienga (Gabon) | [31] | 69.5 | **80.2** | **80.2** | **80.2** | **80.2** | **84.8** | **100** |
| Kilifi (Kenya) | [32] | 67.7 | **78.2** | **79.4** | **79.4** | 79.4 | **84.7** | **96.7** |
| Kisian (Kenya) | [33] | 39.9 | 62.8 | 71.3 | **71.3** | 71.3 | **83.1** | **91.5** |
| Sarididi (Kenya) | [33] | 40.7 | 61.4 | 66.0 | 66.0 | 66.0 | 70.7 | **91.9** |
| Matola (Mozambique) | [34] | 42.3 | 64.1 | 72.2 | **72.2** | 72.2 | **80.6** | **94.8** |
| Bo District (Sierra Leone) | [35] | 63.9 | **83.3** | **90.0** | **90.0** | **90.0** | **93.4** | **97.0** |
| Bagamoyo (Tanzania) | [36] | **85.7** | **90.9** | **93.0** | **93.0** | **93.0** | **93.7** | **97.4** |
| ^¥^Results shown in bold indicate sites fulfilling each of the proposed definitions. Definition A (75% or more of the episodes occurring in 6 or less months of the year), Definition B (≥75% of episodes in ≤3 months), Definition C (≥75% of episodes in ≤5 months), Definition D (≥75% of episodes in ≤7 months), Definition E (≥75% of episodes in ≤9 months), Definition F (≥70% of episodes in ≤6 months), and Definition G (≥80% of episodes in ≤6 months).  ^∫^ Several sites had monthly malaria data for more than one calendar year and these were analysed separately. | | | | | | | | |

**References**

1. Bonnet S, Paul RE, Gouagna C, Safeukui I, Meunier JY, Gounoue R, Boudin C: **Level and dynamics of malaria transmission and morbidity in an equatorial area of South Cameroon**. *Trop Med Int Health* 2002, **7**:249-256.

2. IPTi-Consortium: **Lambarene (Gabon) IPTi trial, pers comm.** In*.*; 2006.

3. Chandramohan D, Owusu-Agyei S, Carneiro I, Awine T, Amponsa-Achiano K, Mensah N, Jaffar S, Baiden R, Hodgson A, Binka F, Greenwood B: **Cluster randomised trial of intermittent preventive treatment for malaria in infants in area of high, seasonal transmission in Ghana**. *BMJ* 2005, **331**:727-733.

4. Bloland PB, Boriga DA, Ruebush TK, McCormick JB, Roberts JM, Oloo AJ, Hawley W, Lal A, Nahlen B, Campbell CC: **Longitudinal cohort study of the epidemiology of malaria infections in an area of intense malaria transmission II. Descriptive epidemiology of malaria infection and disease among children**. *Am J Trop Med Hyg* 1999, **60**:641-648.

5. Sissoko MS, Dicko A, Briet OJ, Sissoko M, Sagara I, Keita HD, Sogoba M, Rogier C, Toure YT, Doumbo OK: **Malaria incidence in relation to rice cultivation in the irrigated Sahel of Mali**. *Acta Trop* 2004, **89**:161-170.

6. Saute F, Aponte J, Almeda J, Ascaso C, Vaz N, Dgedge M, Alonso P: **Malaria in southern Mozambique: incidence of clinical malaria in children living in a rural community in Manhica district**. *Trans R Soc Trop Med Hyg* 2003, **97**:655-660.

7. Macete E, Aide P, Aponte JJ, Sanz S, Mandomando I, Espasa M, Sigauque B, Dobano C, Mabunda S, Dgedge M, Alonso P, Menendez C: **Intermittent preventive treatment for malaria control administered at the time of routine vaccinations in mozambican infants: a randomized, placebo-controlled trial**. *J Infect Dis* 2006, **194**:276-285.

8. Cox MJ, Kum DE, Tavul L, Narara A, Raiko A, Baisor M, Alpers MP, Medley GF, Day KP: **Dynamics of malaria parasitaemia associated with febrile illness in children from a rural area of Madang, Papua New Guinea**. *Trans R Soc Trop Med Hyg* 1994, **88**:191-197.

9. Charlwood JD, Qassim M, Elnsur EI, Donnelly M, Petrarca V, Billingsley PF, Pinto J, Smith T: **The impact of indoor residual spraying with malathion on malaria in refugee camps in eastern Sudan**. *Acta Trop* 2001, **80**:1-8.

10. Kitua AY, Smith T, Alonso PL, Masanja H, Urassa H, Menendez C, Kimario J, Tanner M: ***Plasmodium falciparum* malaria in the first year of life in an area of intense and perennial transmission**. *Trop Med Int Health* 1996, **1**:475-484.

11. Alonso PL, Smith T, Armstrong Schellenberg JRM, Masanja H, Mwankusye S, Urassa H, Bastos de Azevedo I, Chongela J, Kobero S, Menendez C, Hurt N, Thomas MC, Lyimo E, Weiss NA, Hayes R, Kitua AY, Lopez MC, Kilama WL, Teuscher T, Tanner M: **Randomised trial of efficacy of SPf66 vaccine against Plasmodium falciparum malaria in children in southern Tanzania**. *Lancet (British edition)* 1994, **344**:1175-1181.

12. Alonso PL, Smith TA, Armstrong-Schellenberg JR, Kitua AY, Masanja H, Hayes R, Hurt N, Font F, Menendez C, Kilama WL, Tanner M: **Duration of protection and age-dependence of the effects of the SPf66 malaria vaccine in African children exposed to intense transmission of Plasmodium falciparum**. *J Infect Dis* 1996, **174**:367-372.

13. Schellenberg D, Menendez C, Kahigwa E, Aponte J, Vidal J, Tanner M, Mshinda H, Alonso P: **Intermittent treatment for malaria and anaemia control at time of routine vaccinations in Tanzanian infants: a randomised, placebo-controlled trial**. *Lancet (British edition)* 2001, **357**:1471-1477.

14. Schellenberg D, Aponte J, Kahigwa E, Mshinda H, Tanner M, Menendez C, Alonso P: **The incidence in children of clinical malaria detected by active case detection in Ifakara, southern Tanzania**. *Trans R Soc Trop Med Hyg* 2003, **97**:1-8.

15. Davis JC, Clark TD, Kemble SK, Talemwa N, Njama-Meya D, Staedke SG, Dorsey G: **Longitudinal study of urban malaria in a cohort of Ugandan children: description of study site, census and recruitment**. *Malar J* 2006, **5**:18.

16. Kaneko A, Taleo G, Kalkoa M, Yaviong J, Reeve PA, Ganczakowski M, Shirakawa C, Palmer K, Kobayakawa T, Bjorkman A: **Malaria epidemiology, glucose 6-phosphate dehydrogenase deficiency and human settlement in the Vanuatu Archipelago**. *Acta Trop* 1998, **70**:285-302.

17. Bernardino L: **Severe Malaria admissions in 2001. Luanda (Angola)**. In*.*; Personal communication (2004).

18. Taylor T, Olola C, Valim C, Agbenyega T, Kremsner P, Krishna S, Kwiatkowski D, Newton C, Missinou M, Pinder M, Wypij D: **Standardized data collection for multi-center clinical studies of severe malaria in African children: establishing the SMAC network**. *Trans R Soc Trop Med Hyg* 2006, **100**:615-622.

19. Brewster DR, Kwiatkowski D, White NJ: **Neurological sequelae of cerebral malaria in children**. *Lancet* 1990, **336**:1039-1043.

20. Jallow M: **Hospital Admissions with Severe Malaria Morbidity in RVTH, Banjul 1996-2002. MalariaGEN project**. In*.*; Personal communication (2004).

21. Snow RW, Armstrong Schellenberg JRM, Peshu N, Forster D, Newton CRJC, Winstanley PA, Mwangi I, Waruiru C, Warn PA, Newbold C, Marsh K: **Periodicity and space-time clustering of severe childhood malaria on the coast of Kenya**. *Trans R Soc Trop Med Hyg* 1993, **87**:386-390.

22. Varandas L, Julien M, Gomes A, Rodrigues P, Van Lerberghe W, Malveiro F, Aguiar P, Kolsteren P, Van Der Stuyft P, Hilderbrand K, Labadarios D, Ferrinho P: **A randomised, double-blind, placebo-controlled clinical trial of vitamin A in severe malaria in hospitalised Mozambican children**. *Ann Trop Paediatr* 2001, **21**:211-222.

23. Gellert S, Hassan BY, Meleh S, Hiesgen G: **Malaria prevalence and outcome in the in-patients of the Paediatric Department of the State Specialists Hospital (SSH), Maiduguri, Nigeria**. *J Trop Pediatrics* 1998, **44**:109-113.

24. Faye O, N'Dir O, Gaye O, Fall M, Diallo S, Billon C: **[Care charges and direct costs related to hospitalization of Senegalese children with cerebral malaria. Study of 76 cases in the Albert-Royer Hospital in Dakar in 1991-1992]**. *Sante* 1995, **5**:315-318.

25. Reyburn H, Mbatia R, Drakeley C, Carneiro I, Mwakasungula E, Mwerinde O, Saganda K, Shao J, Kitua A, Olomi R, Greenwood BM, Whitty CJ: **Overdiagnosis of malaria in patients with severe febrile illness in Tanzania: a prospective study**. *BMJ* 2004, **329**:1212.

26. Schellenberg D, Menendez C, Aponte J, Guinovart C, Mshinda H, Tanner M, Alonso P: **The changing epidemiology of Malaria in Ifakara Town, southern Tanzania**. *Trop Med Int Health* 2004, **9**:68-76.

27. Thuma PE: **Hospital Admissions of Severe Malaria Morbidity in Macha 2003-2004**. In*.*; Personal communication (2004).

28. Antonio-Nkondjio C, Awono-Ambene P, Toto JC, Meunier JY, Zebaze-Kemleu S, Nyambam R, Wondji CS, Tchuinkam T, Fontenill D: **High malaria transmission intensity in a village close to Yaounde, the capital city of Cameroon**. *J Med Entomol* 2002, **39**:350-355.

29. Dossou-yovo J, Doannio JM, Riviere F, Chauvancy G: **Malaria in Cote d'Ivoire wet savannah region: the entomological input**. *Trop Med Parasitol* 1995, **46**:263-269.

30. Elissa N, Karch S, Bureau P, Ollomo B, Lawoko M, Yangari P, Ebang B, Georges AJ: **Malaria transmission in a region of savanna-forest mosaic, Haut-Ogooue, Gabon**. *J Am Mosq Control Assoc* 1999, **15**:15-23.

31. Elissa N, Migot-Nabias F, Luty A, Renaut A, Toure F, Vaillant M, Lawoko M, Yangari P, Mayombo J, Lekoulou F, Tshipamba P, Moukagni R, Millet P, Deloron P: **Relationship between entomological inoculation rate, *Plasmodium falciparum* prevalence rate, and incidence of malaria attack in rural Gabon**. *Acta Trop* 2003, **85**:355-361.

32. Mbogo CNM, Snow RW, Khamala CPM, Kabiru EW, Ouma JH, Githure JI, Marsh K, Beier JC: **Relationships between *Plasmodium falciparum* transmission by vector populations and the incidence of severe disease at nine sites on the Kenyan coast**. *Am J Trop Med Hyg* 1995, **52**:201-206.

33. Beier JC, Perkins PV, Onyango FK, Gargan TP, Oster CN, Whitmire RE, Koech DK, Roberts CR: **Characterization of malaria transmission by Anopheles (Diptera: Culicidae) in western Kenya in preparation for malaria vaccine trials**. *J Med Entomol* 1990, **27**:570-577.

34. Mendis C, Jacobsen JL, Gamage-Mendis A, Bule E, Dgedge M, Thompson R, Cuamba N, Barreto J, Begtrup K, Sinden RE, Hogh B: ***Anopheles arabiensis* and *An. funestus* are equally important vectors of malaria in Matola coastal suburb of Maputo, southern Mozambique**. *Med Vet Entomol* 2000, **14**:171-180.

35. Bockarie MJ, Service MW, Barnish G, Maude GH, Greenwood BM: **Malaria in a rural area of Sierra Leone. III. Vector ecology and disease transmission**. *Ann Trop Med Parasitol* 1994, **88**:251-262.

36. Shiff CJ, Minjas JN, Hall T, Hunt RH, Lyimo S, Davis JR: **Malaria infection potential of anopheline mosquitoes sampled by light trapping indoors in coastal Tanzanian villages**. *Med Vet Entomol* 1995, **9**:256-262.
